# Update of the EMQN/ACGS best practice guidelines for molecular analysis of Prader-Willi and Angelman syndromes

**DOI:** 10.1038/s41431-019-0435-0

**Published:** 2019-06-24

**Authors:** Jasmin Beygo, Karin Buiting, Simon C. Ramsden, Rachael Ellis, Jill Clayton-Smith, Deniz Kanber

**Affiliations:** 1 Institut für Humangenetik, Universitätsklinikum Essen, Universität Duisburg-Essen, Essen, Germany; 2 Manchester Centre for Genomic Medicine, Manchester University Hospitals NHS Foundation Trust, Manchester Academic Health Sciences Centre, Manchester, UK; 3 Department of Medical Genetics, Yorkhill NHS Trust, Yorkhill Hospital, Glasgow, G3 8SJ UK; 4 Division of Evolution and Genomic Sciences School of Biological Sciences University of Manchester, Manchester, UK

**Keywords:** Diseases, Development

## Abstract

This article is an update of the best practice guidelines for the molecular analysis of Prader-Willi and Angelman syndromes published in 2010 in BMC Medical Genetics [1]. The update takes into account developments in terms of techniques, differential diagnoses and (especially) reporting standards. It highlights the advantages and disadvantages of each method and moreover, is meant to facilitate the interpretation of the obtained results - leading to improved standardised reports.

## Clinical background

Prader-Willi syndrome (PWS, #176270) is characterised by severe hypotonia and feeding difficulties in early infancy, followed in later infancy or early childhood by excessive eating and gradual development of morbid obesity (unless eating is controlled by dietary restriction or behaviour modification). Motor milestones and language development are delayed. All individuals have some degree of cognitive impairment, although some will have an IQ within the normal range. A distinctive behavioural phenotype (with temper tantrums, stubbornness, manipulative behaviour, and obsessive-compulsive characteristics) is common. Hypogonadism is present in both males and females, and manifests as genital hypoplasia, incomplete pubertal development, and in most, infertility. Short stature is common; characteristic facial features, strabismus, and scoliosis are often present, and non-insulin-dependent diabetes mellitus often occurs in obese individuals. Consensus diagnostic clinical criteria for PWS have been developed [2, 3], but genetic testing is necessary for confirmation of the clinical diagnosis and for determination of the recurrence risk.

Angelman syndrome (AS, #105830) is characterised by severe developmental delay, absent or severely limited speech, gait ataxia and/or tremulousness of the limbs, and a unique behaviour with a happy demeanour that includes frequent and sometimes inappropriate laughter, smiling, and excitability. In addition, microcephaly and seizures are common. Affected individuals usually have a characteristic electroencephalography (EEG) appearance with striking high-voltage activity. Developmental delay is first noted at around 6 months of age; however, the unique clinical features of AS may not manifest until after one year of age, and it can take several years before the correct clinical diagnosis is obvious. Consensus clinical diagnostic criteria have also been developed for AS [4, 5]. Again, for confirmation of the clinical diagnosis and determination of the recurrence risk genetic testing is required.

## Genetic background

**Fig. 1:**
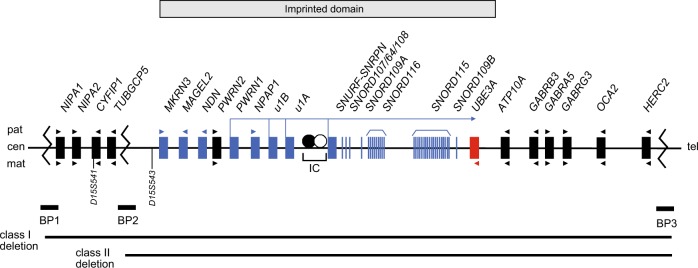
Overview of the imprinted region 15q11q13. Schematic overview of the genes located within the PWS and AS critical region on chromosome 15q11q13. Upper part - paternal allele (pat), lower part - maternal allele (mat). Blue boxes/vertical lines represent paternally expressed genes/snoRNAs; red box, maternally expressed gene; black boxes, biallelically expressed genes and arrow heads represent the orientation of transcription. IC imprinting centre, BP common breakpoint cluster region, cen centromeric, tel telomeric. Modified from Ramsden et al. [1]

The proximal long arm of human chromosome 15 (15q11q13) contains a cluster of imprinted genes, which are under the control of an imprinting centre (IC) [6]. Some of these genes are expressed from the paternal or maternal chromosome only. PWS arises from the loss of function of paternally expressed genes in 15q11q13. This loss can be a result of either a large deletion of this region on the paternal allele that is in most cases de novo, maternal uniparental disomy (UPD) of chromosome 15 or the silencing of the paternal allele by an imprinting defect (ID) on the paternal chromosome [7]. Such an ID can be due to an IC deletion (see below) or occur without an underlying change in the DNA sequence. So far, several genes preferentially or exclusively expressed from the paternal chromosome have been described: *MKRN3, MAGEL2, NDN, PWRN1, NPAP1 (previously C15orf2), SNURF-SNRPN* and several C/D box small nucleolar RNA (snoRNA) genes (see Fig. 1). At least three of these genes *SNRPN, MAGEL2* and *NDN*, have differentially methylated CpG islands in their promoter regions that are methylated on the maternal chromosome leading to silencing of the maternal allele (new nomenclature: *SNURF*:TSS-DMR, *MAGEL2*:TSS-DMR and *NDN*:TSS-DMR [8]). The *SNURF*:TSS-DMR is a primary DMR where the methylation imprint is already set in the female germline. On the other hand, the *MAGEL2*:TSS-DMR and the *NDN*:TSS-DMR are secondary DMRs where the methylation imprint is set postzygotically (see section “Prenatal diagnosis”, Supplementary Fig. 1).

Whereas most of the snoRNA genes are present as single genes (*SNORD64, SNORD107, SNORD108, SNORD109A* and *SNORD109B*), the two snoRNA genes *SNORD116* and *SNORD115* are present in 24 and 47 gene copies, respectively. It has been demonstrated that deficiency of *SNORD116* snoRNAs causes the key characteristics of the PWS phenotype [9–12], however one or more additional genes in the region are likely to contribute.

AS is caused by loss of function of the maternally expressed gene *UBE3A* in neuronal cells. The major disease mechanisms are similar to those in PWS being either a large deletion of 15q11q13 on the maternal chromosome that is in most cases de novo, a paternal UPD or an ID affecting the maternal chromosome [13, 14]. Again, the ID can arise due to an IC deletion (see below) or without an underlying change in the DNA sequence. Furthermore, in more than 40% of patients with AS and an ID without an IC deletion, the ID is present in a subset of cells only (somatic mosaicism), indicating that it occurred after fertilisation [14, 15]. Those patients sometimes present with a phenotype resembling that of PWS. On the other hand in PWS only very rare patients have been described with a mosaic ID [16, 17].

In addition, disease causing variants (point mutations) in the imprinted *E6-AP ubiquitin-protein ligase* gene (*UBE3A*) cause AS [18–20]. Imprinted *UBE3A* expression is restricted to brain cells where expression is exclusively from the maternal chromosome and disruption of expression of this gene is the major cause of the disease. In contrast to PWS, ~15% of patients with the clinical diagnosis of AS have a genetic defect of unknown aetiology.

The IC has been defined using microdeletions in a small number of patients with PWS or AS and an ID, who have small, atypical deletions. It has a bipartite structure with two critical regions, the AS-SRO (shortest region of deletion overlap; AS-IC element) and the PWS-SRO (PWS-IC element [6]). By analysing a very large series of PWS and AS patients with an ID it has been shown that the vast majority of IDs have occurred spontaneously in the absence of DNA sequence changes [16]. In particular, disease causing variants within the IC have not been detected to date [16, 21, 22]. Nevertheless, the presence or absence of an IC-deletion has to be determined as the recurrence risk is 50% in case of a familial IC-deletion.

A summary of the causative genetic mechanisms and recurrence risks in PWS and AS is given in Tables 1 and 2.

**Table 1:** Molecular defects and recurrence risks in PWS

Genetic defect	Proportion of cases [7]	Recurrence risk
De novo deletion of 15q11q13 on the paternal chromosome	70–75%	<1%^a^
Maternal uniparental disomy (UPD) of chromosome 15	25–30%	<1%^b^
Imprinting defects (without an imprinting centre deletion)	1%	<1%
Imprinting centre deletion	≈10–15% of patients with an imprinting defect	50% (if present in a non-mosaic state in the father)

^a^If paternal karyotype is normal

^b^If parental karyotypes are normal

**Table 2:** Molecular defects and recurrence risks in AS

Genetic defect	Proportion of cases [13]	Recurrence risk
De novo deletion of 15q11q13 on the maternal chromosome	75%	<1%^a^
Paternal uniparental disomy (UPD) of chromosome 15	1–2%	<1%^b^
Imprinting defect (without an imprinting centre deletion)	3%	<1%
Imprinting centre deletion	≈10–15% of patients with an imprinting defect	50% (if present in a non-mosaic state in the mother)
*UBE3A* mutation	5–10%	50% (if present in a non-mosaic state in the mother)
No identifiable molecular abnormality	10–15%	Unknown (up to 50%)

^a^If maternal karyotype is normal

^b^If parental karyotypes are normal

There are a number of molecular genetic approaches to confirm these two disorders. The most common is DNA-based methylation testing to detect hyper- or hypomethylation within the PWS and AS critical region. This will detect more than 99% of individuals with PWS and ~80% of individuals with AS. *UBE3A* sequence analysis detects disease causing variants in approximately a further 5–10% of individuals with AS, however *UBE3A* analysis is not considered further in this article.

## Methods

The original guidelines for PWS and AS were assessed by consideration of the external quality assessment returns submitted to the European Molecular Genetics Quality Network (EMQN) and the United Kingdom External Quality Assessment Scheme (UK NEQAS) for Molecular Genetics over a 5-year period. The guidelines were posted on the web-site of the UK Clinical Molecular Genetics Society (CMGS; subsequently renamed The Association for Clinical Genomic Science (ACGS) since 2016) for consultation and amendment between 19 May 2008 and 6 January 2010 and heads of the constituent laboratories were invited to comment. In the light of feedback amendments were made and the final document was ratified by the CMGS Executive Committee on 10 January 2010. In addition they were approved by the European Molecular Genetics Quality Network (EMQN) Steering Group on 22 January 2010.

The updated version in this article was available through the EMQN for consultation and amendment by the community of 105 laboratories participating in the EMQN-organised scheme for PW/AS, between 20 June 2018 and 13 July 2018. The feedback was collected, evaluated and the draft document amended accordingly.

## Results

### Strategies for the analysis of PWS and AS

The approach to the laboratory diagnosis of PWS and AS depends on many factors, including the availability of samples, the arrangement of laboratory services and the patterns of referral. The most sensitive single approach to diagnosing PWS and AS is to study the methylation within 15q11q13 using molecular genetic techniques. These will detect deletions, UPD and ID by establishing either hypermethylation/complete methylation (PWS) or hypomethylation/complete absence of methylation (AS). Note that for AS mosaic cases only a partial hypomethylation is detected. Broadly speaking, methylation studies take one of two forms:

(i) The simultaneous assessment of methylation status and genomic dosage at numerous sites across the 15q11q13 region, by the use of methylation-sensitive multiplex ligation-dependent probe amplification (MS-MLPA). This approach will confirm the diagnosis and further identify the presence of a causative deletion (large deletion or deletion of the imprinting centre (IC-deletion)). In the absence of a deletion methylation results alone are not sufficient to discriminate between UPD and ID. Therefore, microsatellite analysis is required.
(ii) The detection of methylation status solely at the *SNRPN* locus by use of methylation-specific PCR (MS-PCR). This approach will confirm a diagnosis but will provide no further information regarding the disease mechanism. Thus, it is still open whether the molecular cause is a large deletion, a UPD or an ID with or without an IC-deletion necessitating follow up studies (dosage analysis by MLPA or FISH, microsatellite analysis, IC analysis (in case FISH was done for the deletion analysis)).

**Fig. 2:**
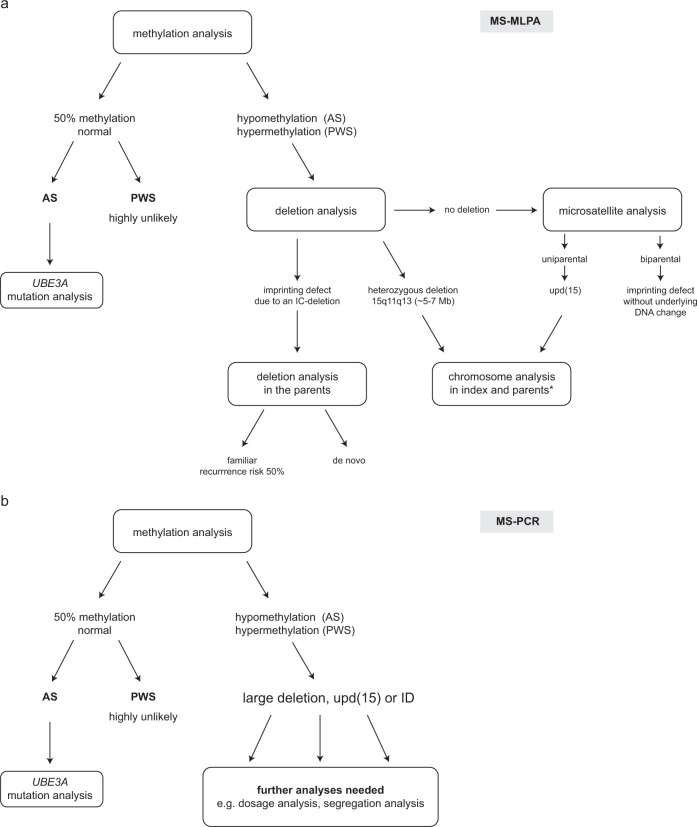
Testing strategies for the molecular analysis of PWS and AS using **a** MS-MLPA and **b** MS-PCR. Note that MS-PCR cannot distinguish between the different molecular causes of AS and PWS. *In case of a upd(15) both parents should be investigated to rule out cytogenetic rearrangements (e.g. Robertsonian translocations). In case of PWS or AS due to a large, heterozygous deletion the father or the mother, respectively should be investigated

It is essential to note that approach (ii) will not distinguish between the molecular causes. MS-MLPA however provides more information in this regard (see below). Figure 2 shows an example of testing strategies for approaches (i) and (ii).

### Molecular genetic testing methods

#### MS-MLPA

MS-MLPA provides a means to simultaneously detect copy number changes and DNA methylation within 15q11q13 in a semi-quantitative manner. Almost all laboratories using this approach make use of a commercially available MS-MLPA kit available from MRC Holland (http://www.mrc-holland.com). The MS-MLPA kit contains copy number probes, specific for sequences in or close to the PWS/AS critical region on 15q11q13 as well as outside the PWS/AS region and on other chromosomes which serve as controls. Among the PWS/AS specific probes, there are methylation-sensitive probes that contain a *Hha*I restriction site. The current MS-MLPA kit ME028-C1 contains five methylation-sensitive probes from the PWS/AS region that represent differentially methylated sites (four for the *SNURF-SNRPN* exon1/promoter region and intron 1 and one for *MAGEL2*), and another one from the completely unmethylated promoter region of *UBE3A*. Moreover, the kit contains additional methylation-sensitive control probes for completely unmethylated sequences from other chromosomes that will indicate complete digestion by the *Hha*I enzyme and therefore serve as digestion control probes.

Dosage analysis by MS-MLPA offers the opportunity to detect large deletions, the most frequent molecular lesions in patients with PWS and AS. In rare cases, a larger deletion can extend telomeric. Another advantage of the MS-MLPA kit is that it will identify IC-deletions in PWS and AS cases with an ID. The kit ME028-C1 contains two probes for the *SNRPN* exon 1/promoter, a region which represents the smallest region of deletion overlap in patients with PWS (PWS-SRO) and an IC-deletion. Furthermore, there are two probes for *SNRPN* exon U5 which can be used to detect IC-deletions in patients with AS and an ID. Both lie in the smallest region of deletion overlap found in patients with AS and an imprinting defect (AS-SRO). Beside IC-deletions, small deletions affecting the *SNORD116* gene region or partly the *UBE3A* gene can be detected as well. For *UBE3A* deletion detection another MLPA kit (P336-B1, MRC Holland) is available that covers each exon. But this is not the subject of these guidelines.

MS-MLPA has become the method of choice in many diagnostic laboratories as it investigates methylation status of single CpGs at several positions inside a DMR, thereby reducing the risk of false-positive or false-negative results due to SNPs or technical problems. Whereas the MS-PCR is restricted to the CpGs at the primer binding site(s).

There appears to be some naturally-occurring variation in dosage and methylation status which must be taken into account when interpreting MS-MLPA results:

(i) For the two most centromeric probes, *NIPA1* and *TUBGCP5*, copy number variation has been observed. This has been attributed to deletions and duplications encompassing these two probes, and complicates the definition of class I and class II deletions.
  The frequency of the BP1-BP2 duplication seems to be around 0.5% in the general population with the deletion being only slightly less frequent [23].
  The phenotypic outcome of these copy number variations is still a matter of debate and especially the deletion has been linked to a wide variety of phenotypic features from developmental delay to congenital heart defects [24–26]. Newer studies point to a (mild) effect in cognition, a low penetrance and a high proportion of variants inherited from normal parents [27, 28]. Therefore, interpretation of these variants should be done with extreme caution.
  Variation in the hybridisation efficiency due to a copy number variation is also observed for the *SNRPN* exon u1B probe.
(ii) SNPs under hybridisation binding sites can influence probe signals. Consequently, laboratories must exercise extreme caution when interpreting results from a single probe.
(iii) Normal variation in the degree of methylation at the *MAGEL2* locus is frequently observed.

For prenatal diagnosis it should be noted that chorionic villi and amniotic fluid samples show a significant hypomethylation at the *MAGEL2* and *NDN* locus (Supplementary Fig. 1; ME028-C1 Description version C1–03; 24 January 2019, MRC Holland). These results suggest that the methylation of the *MAGEL2* and *NDN* locus is not fully established in chorionic tissue. It is therefore recommended that only the methylation status of the *SNRPN* locus is considered in the context of prenatal diagnosis.

It should be noted that the current commercially available MS-MLPA kit ME028 is not CE-IVD certified for diagnostic use and must be fully validated in individual laboratories prior to implementation. We recommend that recurrent variation observed in the MRC MS-MLPA kit is reported to the manufacturer to facilitate future kit development.

#### MS-PCR

This is based on sodium bisulphite treatment of DNA followed by PCR using primers specific for differentially methylated sites within the *SNRPN* exon 1/promoter regions. Two approaches have been shown to work reliably in interlaboratory comparisons: Kubota et al. [29] describe the use of two primer pairs that can be used separately (simplex PCR) or in combination (duplex PCR). However, it is strongly recommended that the primers are not used in a simplex reaction as this has been shown to result in spurious DNA amplification and/or misdiagnosis resulting from PCR failure [30]. Zeschnigk et al. [31] describe an alternative assay with one common primer that anneals to both alleles and one specific primer each for the methylated and the unmethylated allele. In this case, the three primers are always used together in one reaction. The *SNRPN* exon 1/promoter region is highly conserved but contains some very rare single nucleotide polymorphisms (SNPs) within the primer binding sites (see Supplementary Fig. 2) that can lead to false results. Such variants can now be found in current open access databases [e.g. GnomAD http://gnomad.broadinstitute.org/].

Another point to consider is the challenging detection of mosaic cases by MS-PCR.

#### Alternative techniques

A small number of laboratories use alternative methods of methylation analysis including PCR following restriction digestion with a methylation-sensitive enzyme [32], melt-curve analysis [33], and pyrosequencing [34]. These techniques have not been widely adopted but have all been used successfully within a diagnostic context. However, like the MS-PCR, these alternative techniques only investigate the methylation status and cannot distinguish between the different molecular causes.

A SNP array analysis for PWS/AS testing is of limited suitability as it will identify only patients with a large deletion or an isodisomy for chromosomes 15. It will miss patients with heterodisomy 15 unless both parents are investigated by SNP array as well and the genotypes are analysed in detail. Be aware that an ID cannot be detected by this technique. Furthermore, the parental origin of the deletion or the UPD (in case parental samples have not been investigated) remains unknown. Consequently, patients who were found to have a deletion or an isodisomy and also patients without evidence for a deletion must be investigated for methylation.

#### Microsatellite analysis (MSA)

When a molecular diagnosis of AS or PWS is confirmed with any of the above techniques microsatellite analysis will often be required to distinguish between a UPD and an ID. In some cases MSA is also used to detect large deletions, if enough informative markers are present. However we would recommend dosage analysis by MLPA as this can immediately detect large and IC-deletions.

At present many microsatellites are available for chromosome 15, and it is outside the scope of this article to provide a comprehensive list of suitable markers. However, it is worth noting the following markers that have been used in the past and are known to be compromised:

- *D15S113*. This is a CA repeat from within the critical region. The presence of null alleles (or non-amplification alleles) has been observed with this marker and can complicate the analysis of AS and PWS cases. Under certain conditions, a non-amplification allele can be misinterpreted as a small deletion. The frequency of these alleles in families without AS has been estimated to be around 4%. Alternative primers can be designed; however, this marker is best avoided.
- *D15S817*. The presence of three alleles has been observed with this marker with certain primer sets due to complex duplications in the region where the marker is located. Since there is a low frequency/density of useful markers for the more centromeric PWS/AS region, the use of the following primer pair (*D15S817F*, 5′-TGGAACCAATAGGATAGACAC-3′ *D15S817R*, 5′-GGTCAGCCTCCATAATCA-3′) can resolve this problem.

For microsatellite analysis we recommend choosing markers within and outside the PWS/AS region and analysing an appropriate number of markers to be able to make a solid interpretation. In some countries it is compulsory to analyse at least eight markers. Moreover, an interpretation should never be built on one single informative marker.

Once the diagnosis of PWS or AS has been confirmed using methylation analysis, the interpretation of the microsatellite results is as follows:

- *Uniparental inheritance both inside and outside the critical region:* In this case, the disease is due to UPD. It is important to note that AS and PWS can be caused by either chromosomal isodisomy or heterodisomy (Meiosis I or Meiosis II non-disjunction). Further, heterodisomy or isodisomy at a single locus does not necessarily reflect the disomy status along the entire chromosome depending on the rate and level of crossing over and the meiotic stage at which non-segregation occurred.
- *Biparental inheritance both inside and outside the critical region:* In this case the disease is due to an ID. Exclusion of an IC-deletion must ensue if not already performed (e.g. by MS-MLPA).
- *Uniparental inheritance inside the critical region, biparental outside:* In this case the disease is most likely due to a deletion of the critical region. Laboratories must not interpret results from a single informative microsatellite without supporting evidence. In very rare cases such a constellation can be seen in patients with a segmental UPD [35].

### Interpretation of diagnostic testing results

#### Normal methylation result

A normal methylation pattern rules out PWS on the basis of most known cases to date (sensitivity ~99%) and around 80% of AS cases.

Despite early reports of possible deletion mosaicism in PWS [36, 37] the case for deletion mosaicism remains unproven [38].

In the case of AS, if the clinical suspicion remains strong with normal methylation then it is recommended to undertake *UBE3A* analysis as mutations in this gene will have a recurrence risk of 50% if the mother is a non-mosaic mutation carrier.

#### Deletions

The de novo interstitial deletion of chromosome 15, del(15)(q11q13), which includes the entire imprinted domain plus several non-imprinted genes, extends ~5–7 Mb and is found in the majority of patients with PWS and AS. In both syndromes, the same region is affected, but in PWS the deletion is always on the paternal chromosome, whereas in AS it is always on the maternal chromosome. This deletion is probably one of the most common pathogenic deletions observed in humans [7]. In a few patients, the region is deleted as the result of an unbalanced translocation. At the molecular level, two classes of deletions (class I and II) can be distinguished. In both classes, the distal breakpoints are close to, but telomeric to the *OCA2* gene (breakpoint region 3, BP3, see Fig. 1). In class I deletions (30–40% of deletion cases), the proximal breakpoint is centromeric to the marker *D15S541* (breakpoint region 1, BP1). In class II deletions (60–70% of deletion cases) the proximal breakpoint is between *D15S541* and *D15S543* (breakpoint region 2, BP2). The clustering of the deletion breakpoints is due to the presence of large duplicated sequence stretches of 200–400 kb in size in the common breakpoint regions that are susceptible to non-homologous crossovers [39–41]. There are rare cases with atypical deletions that can range until BP4/BP5 [42]. Cases of large deletions should be further investigated by cytogenetic analysis to rule out the presence of (very rare) balanced cytogenetic rearrangements in the appropriate parent (father for PWS and mother for AS) that may predispose to a deletion.

#### UPD

The second most frequent finding in PWS is maternal UPD (upd(15)mat; nomenclature according to ISCN 2016 [43]) of chromosome 15. These patients have two maternal copies of chromosome 15 and lack a paternal copy. As the paternally expressed genes are silent on the maternal chromosome, upd(15)mat is associated with a complete loss of function of these genes. The reciprocal finding is made in 1–2% of patients with AS. These patients have two paternal copies of chromosome 15 and lack a maternal copy (upd(15)pat). In brain cells *UBE3A* is silent on the paternal chromosome, so upd(15)pat is associated with a complete loss of function of this gene in this tissue. UPD can arise as a result of meiotic and/or mitotic errors [44]. Mechanisms include trisomy rescue, monosomy rescue, complementation and postfertilisation error. During meiosis, the diploid set of chromosomes (*n* **=** 46) is reduced to a haploid set (*n* **=** 23). Nondisjunction of the homologous chromosomes 15 during, e.g., female meiosis I or nondisjunction of the two sister chromatids during female meiosis II results in an oocyte with two chromosomes 15 or no chromosome 15. In these cases, fertilisation by a sperm with one chromosome 15 will result in a zygote which is trisomic or monosomic for chromosome 15 respectively. These conditions are not compatible with normal development, but can be ‘rescued’ by loss of one chromosome 15 from a trisomic cell or duplication of the paternal chromosome 15 in a monosomic cell. With trisomy rescue, in two-thirds of cases, one of the two maternal chromosomes will be lost from the trisomic cell, resulting in a normal set of chromosomes. If, however, the paternal chromosome is lost, the cell is left with two maternal chromosomes 15 (upd(15)mat; PWS). Duplication of the paternal chromosome 15 in a monosomic cell will lead to upd(15)pat (AS).

Alternative mechanisms for UPD such as complementation involving both a nullisomic and disomic gamete or rescued paternal 15 trisomy have also been reported, however are considered rare [45].

Cases of UPD should be further investigated by cytogenetic analysis to rule out the presence of a (very rare) balanced Robertsonian translocation in one of the parents - both parents have to be analysed irrespective of a detected upd(15)mat or upd(15)pat.

#### Imprinting defects

There are a small number of patients with either PWS (1%) or AS (3%) that show a hypermethylation/complete methylation or hypomethylation/complete absence of methylation, respectively, without a large deletion of 15q11q13. Moreover, they show biparental inheritance for chromosome 15 markers, both inside and outside the critical region. These patients have an ID. The majority of these patients are sporadic cases without any detectable deletion or a DNA sequence change at the IC region. However, in 10–15% of cases, the ID is caused by an IC-deletion. In most cases, the IC-deletion is familial and thus associated with a 50% recurrence risk. In some cases the IC-deletion is de novo or a consequence of germ line mosaicism in the father or the mother. In these families, the recurrence risk ranges from 0–50%, depending on the degree of the mosaicism in the germ line [16]. An IC-deletion is the only kind of DNA sequence change found in patients with an ID with the exception of a single case where a familial inversion has been identified which disrupts the IC region [46].

If a laboratory does not use an assay capable of assessing copy number in the AS- and PWS-SROs then, in the case of a confirmed diagnosis where a common deletion or UPD has been excluded, it is essential that the family workup is completed in a laboratory able to undertake this test. On confirmation of an IC-deletion parents should also be tested if available (father in case of PWS and mother in case of AS) to establish recurrence risks in the family.

As mentioned before, more than 40% of AS imprinting defect patients without an IC-deletion have a partial hypomethylation and therefore are somatic mosaics for an ID (the detected degree of hypomethylation depends on the number of cells carrying the ID). In these cases the ID occurred after fertilisation and is not associated with an increased recurrence risk [15].

### Reporting

Some general considerations for the reports:

- It is recommended that laboratories do not use joint PWS/AS report templates.
- It is essential to state clearly on the report the method(s) used to carry out genetic analysis, with an appropriate reference; for example, “ME028-C1 (MRC Holland)”, always including the version number or “Zeschnigk et al. 1997 Eur J Hum Genet, 5:94-99”.
- Identifier of the patient and of all other family members from whom a sample was used for diagnostics, e.g. in MSA, must be given.
- The source of the DNA sample should be given on the report, as it can be important for example in case of prenatal diagnosis.
- Results should be reported in a clear and concise fashion. The interpretation should be given separately and not be merged with the results.
- It should be stated clearly that ‘the diagnosis of PWS or AS is confirmed’. Phrasings like ‘is consistent with’, ‘supports the diagnosis’, ‘is in accordance with’, ‘are compatible with’ are ambiguous.
- Reports should inform about the recurrence risk.
- Genetic counselling should be offered to all families irrespective of whether a diagnosis is confirmed or not (different standards may apply in different countries).
- Limitations and test sensitivity should be mentioned if diagnosis could not be confirmed (e.g. low grade mosaicism might escape detection).
- Inadequate terms to describe methylation results should be avoided. For the description of the result obtained terms as ‘PWS pattern’, ‘abnormal methylation pattern’, ‘change in methylation’ or ‘pathological result’ are not appropriate. The best is to describe what is observed. Examples for such descriptions including adequate terms of the methylation result are given below.

More specific recommendations are as follows:

### Diagnostic referral for PWS (MS-MLPA)

(i) Normal methylation and normal gene dosage at 15q11q13 detected:
  This result excludes a large deletion on the paternal allele, a UPD of chromosome 15 and an ID. A diagnosis of PWS is highly unlikely (sensitivity of the test ~99%).
(ii) Heterozygous deletion and hypermethylation/complete methylation detected:
  Gene dosage analysis shows a 50% reduction for # probes within the chromosomal region 15q11q13 (33 of probes from *TUBGCP5* to *OCA2*, class I deletion; 31 of probes from *MKRN3* to *OCA2*, class II deletion; number of probes depending on the kit version used, here ME028-C1). The methylation analysis shows a hypermethylation/complete methylation at all five methylation-sensitive probes, indicating that the deletion is on the paternal allele.
  This result confirms the diagnosis of PWS. The molecular cause is a heterozygous deletion within 15q11q13 (class I or class II deletion) on the paternal allele.
  Cytogenetic analysis in the patient and the father should be performed to rule out the presence of cytogenetic rearrangements which would increase the recurrence risk.
(iii) Normal gene dosage and hypermethylation/complete methylation detected:
  Normal gene dosage, but a hypermethylation/complete methylation at all five methylation-specific probes. This result confirms the diagnosis of PWS. The molecular cause of PWS is either a maternal UPD of chromosome 15 or an ID.
  Microsatellite analysis should be performed in the family to differentiate between a upd(15)mat and an ID.
  If a upd(15)mat is confirmed subsequent cytogenetic analyses should be performed in the patient and the parents to rule out cytogenetic rearrangements which would increase the recurrence risk.
  If an ID is confirmed the recurrence risk is not increased, since an IC-deletion has been excluded.
(iv) Heterozygous deletion of the IC and hypermethylation/complete methylation detected:
  Gene dosage analysis shows a 50% reduction for # probes located in the IC (number and names of probes according to the kit version used). The methylation analysis shows a hypermethylation/complete methylation at all five methylation-specific probes, indicating that the IC-deletion is on the paternal allele.
  This result confirms the diagnosis of PWS. The molecular cause of PWS is an ID due to an IC-deletion.
  MS-MLPA analysis in the father should be recommended to estimate the recurrence risk. If the father is a non-mosaic carrier of the IC-deletion the recurrence risk is 50%. If he has no detectable deletion in blood DNA this does not exclude germ line mosaicism, which is also associated with an increased recurrence risk (see section “Imprinting defect”).

If appropriate other family members should be investigated, too.

### Diagnostic referral for PWS (using MS-PCR or a comparable alternative technique)

(i) Normal methylation at 15q11q13 detected:
  This result excludes a large deletion on the paternal 15q11q13 allele, a UPD of chromosome 15 and an ID. A diagnosis of PWS is highly unlikely (sensitivity of the test ~99%).
(ii) Hypermethylation/complete methylation detected:
  No paternal, unmethylated band detected in the methylation analysis at 15q11q13. This result confirms the diagnosis of PWS. The underlying molecular cause can be either a large deletion of 15q11q13 on the paternal allele, a maternal UPD of chromosome 15 or an ID (with or without an IC-deletion). Further molecular genetic testing (e.g. dosage analysis) is necessary.
  As MS-PCR or equivalent methods that investigate only the methylation status cannot differentiate between the possible molecular genetic mechanisms underlying the PWS diagnosis, additional investigations regarding gene dosage and segregation of chromosome 15 have to be requested. This is of utmost importance as the recurrence risk cannot be determined without knowledge of the underlying molecular genetic cause.

### Diagnostic referral for AS (MS-MLPA)

(i) Normal methylation and normal gene dosage at 15q11q13 detected:
  This result excludes a large deletion on the maternal allele, a UPD of chromosome 15 and an ID.
  This result does not exclude a diagnosis of AS. Analysis of *UBE3A* should ensue if the clinical re-assessment of the patient still suggests AS.
(ii) Heterozygous deletion and hypomethylation/complete absence of methylation detected:
  Gene dosage analysis shows a 50% reduction for # probes within the chromosomal region 15q11q13 (33 of probes from *TUBGCP5* to *OCA2*, class I; 31 of probes from *MKRN3* to *OCA2*, class II; number of probes depending on the kit version used, here ME028-C1). The methylation analysis shows a hypomethylation/complete absence of methylation at all five methylation-specific probes, indicating that the deletion is on the maternal allele.
  This result confirms the diagnosis of AS. The molecular cause is a heterozygous deletion within 15q11q13 (class I or class II deletion) on the maternal allele.
  Cytogenetic analysis in the patient and the mother should be performed to rule out the presence of cytogenetic rearrangements which would increase the recurrence risk.
(iii) Normal gene dosage and hypomethylation/complete absence of methylation detected:
  Normal gene dosage, but a hypomethylation/complete absence of methylation at all five methylation-specific probes. This result confirms the diagnosis of AS. The molecular cause of AS is either a paternal UPD of chromosome 15 or an ID.
  Microsatellite analysis should be performed in the family to differentiate between a upd(15)pat and an ID.
  If a upd(15)pat is confirmed subsequent cytogenetic analyses should be performed in the patient and the parents to rule out cytogenetic rearrangements which would increase the recurrence risk.
  If an ID is confirmed the recurrence risk is not increased, since an IC-deletion has been excluded.
(iv) Normal gene dosage and partial hypomethylation/partial absence of methylation detected:
  Normal gene dosage, but a partial hypomethylation/partial absence of methylation at all five methylation-specific probes. This result confirms the diagnosis of AS. The partial hypomethylation/partial absence of methylation indicates that a somatic mosaic is present where cells with a normal imprint and cells with either a paternal UPD of chromosome 15 or an ID are present.
  Microsatellite analysis should be performed in the family to differentiate between these two possibilities.
(v) Heterozygous deletion of the IC and hypomethylation/complete absence of methylation detected:
  Gene dosage analysis shows a 50% reduction for # probes located in the IC (number and names of probes according to the kit version used). The methylation analysis shows a hypomethylation/complete absence of methylation at all five methylation-specific probes, indicating that the IC-deletion is on the maternal allele.
  This result confirms the diagnosis of AS. The molecular cause of AS is an ID due to an IC-deletion.
  MS-MLPA analysis in the mother should be performed to estimate the recurrence risk. If the mother is a non-mosaic carrier of the IC-deletion the recurrence risk is 50%. However, an absence of the deletion in blood does not exclude a germ line mosaicism which is associated with an increased recurrence risk (see section “Imprinting defect”).

If appropriate other family members should be investigated, too.

### Diagnostic referral for AS (using either MS-PCR or a comparable alternative technique)

(i) Normal methylation at 15q11q13 detected:
  This result excludes a large deletion on the maternal allele, a UPD of chromosome 15 and an ID.
  This result does not exclude a diagnosis of AS. Analysis of *UBE3A* should be performed if the clinical re-assessment of the patient still suggests AS.
(ii) Hypomethylation/complete absence of methylation detected:
  No maternal, methylated band detected in the methylation analysis at 15q11q13. This result confirms the diagnosis of AS. The underlying molecular cause can be either a large deletion of 15q11q13 on the maternal allele, a paternal UPD of chromosome 15 or an ID with or without an IC-deletion. Further molecular genetic testing (e.g. dosage analysis) is necessary.
(iii) Partial hypomethylation/partial absence of methylation detected:
  A faint maternal, methylated band detected in the methylation analysis at 15q11q13. This result confirms the diagnosis of AS. The underlying molecular cause can be either a large deletion of 15q11q13 on the maternal allele, a paternal UPD of chromosome 15 or an ID each in a mosaic state. Most likely are a UPD and an ID. Further molecular genetic testing (e.g. microsatellite analysis) is necessary.

As MS-PCR or equivalent methods that investigate only the methylation status cannot differentiate between the possible molecular genetic mechanisms underlying the AS diagnosis, additional investigations regarding gene dosage and segregation of chromosome 15 have to be requested. This is of utmost importance as the recurrence risk cannot be determined without knowledge of the underlying molecular genetic cause.

### Prenatal diagnosis

When the diagnosis has been confirmed and the molecular cause has been established in the index patient, prenatal diagnosis can be offered to the family. In case of a de novo deletion or UPD the recurrence risk is very low given that the parental chromosomes are normal. Recurrence risk for IDs without an IC-deletion is low. However, prenatal testing can be offered to the families for reassurance regardless of the underlying molecular cause. For cases of familial deletions of the IC prenatal diagnosis should be offered as the recurrence risk is high (50%). Methylation status at *SNRPN* exon 1 is established early in embryonic development and testing DNA extracted from both amniotic cells and chorionic villi (both native and cultured) has been shown to give reliable results [47]. The methylation at the *NDN* locus should not be interpreted in case of prenatal testing. The methylation at the *MAGEL2* locus is more similar to the methylation status at the *SNRPN* region than *NDN*, but may not always be fully established at time of sampling, and is therefore not reliable and should also not be interpreted in case of prenatal testing (ME028-C1 Description version C1-03; 24 January 2019, MRC Holland; Supplementary Fig. 1).

For cases of ID due to IC-deletions or disease causing variants in *UBE3A* in AS cases that are either de novo or a consequence of germ line mosaicism in the parent, the recurrence risk is difficult to predict but may be as high as 50%. In cases of sporadic ID with no detectable deletion, the recurrence risk appears to be low, however since the possibility of recurrence cannot be excluded a prenatal diagnosis should be offered.

### Differential diagnoses

#### Prader-Willi syndrome

Diagnoses which need to be considered in infants with hypotonia include congenital myopathies, central and peripheral neuromuscular disorders, especially type 1 spinal muscular atrophy and the congenital form of myotonic dystrophy. Furthermore anomalies of the central nervous system and peroxisomal disorders should also be ruled out if chromosome 15 methylation is normal [7, 48].

A further phenotype which presents with neonatal hypotonia and short stature together with later-onset obesity is Temple syndrome, an imprinting disorder which can be caused by deletions, ID and upd(14)mat involving the chromosomal region 14q32 [49, 50].

When considering the differential diagnosis of older children with learning disability and obesity, Cohen syndrome, Borjesson-Forssman-Lehman syndrome (males), Bardet-Biedl syndrome, Alstrom syndrome and Fragile X syndrome along with chromosomal disorders including diploid/triploid mosaicism, 1p36 microdeletion syndrome and 16p11.2 deletion syndrome, should be considered [51]. In many of these cases the facial phenotype differs from PWS [52, 53].

Truncating disease causing variants of the imprinted *MAGEL2* gene on 15q11q13 have been described in patients with Schaaf-Yang syndrome (#615547). The phenotypic overlap with PWS includes neonatal hypotonia, intellectual disability and later onset of obesity. Contractures of interphalangeal joints can serve as a distinguishing feature [54, 55]. Only disease causing variants on the paternal allele are causative so that a silent transmission over several generations is possible. Deletions of the whole gene have been reported, but do not lead to PWS or a similar phenotype [56, 57].

#### Angelman syndrome

Around 10–15% of patients with a clinical diagnosis of AS have no detectable disturbance at 15q11q13 using the techniques described here. In rare cases, these patients may be low grade mosaics for an imprinting defect. However, it is more likely that there is an alternative clinical diagnosis and a careful review of the patient’s history, clinical features and EEG findings is recommended. One diagnosis which should be considered in girls is Rett syndrome caused by haploinsufficiency of the *MECP2* gene located on chromosome Xq (male Rett syndrome is rare but possible) [58, 59]. It is extremely difficult to distinguish between Rett syndrome and AS during infancy when both can present with acquired microcephaly, ataxia and frequent smiling. Later, Rett syndrome may be distinguished by the presence of a history of developmental regression, the emergence of stereotypic hand-wringing movements, bouts of hyperventilation and the presence of vasomotor disturbance. If there is a very early onset of seizures, within the first few months, disease causing variants within the *CDKL5* gene should be considered [60]. Mowat-Wilson syndrome, caused by disease causing variants in the *ZEB2* gene on chromosome 2q, is associated with severe learning disability, limited speech, seizures and characteristic facial features that resemble those of AS. In addition Hirschsprung disease, congenital cardiac defects and agenesis of the corpus callosum may be associated with disease causing variants in *ZEB2*. A strong indication is the characteristic appearance of the ear lobes which are upturned and look like “shell pasta”. Pitt-Hopkins Syndrome (PHS) is a sporadic condition caused by disease causing variants or deletions of the *TCF4* gene on chromosome 18q. Patients present with absent speech, seizures and facial features resembling AS, together with a sociable personality. The facial appearance in PHS coarsens with age and the lips in particular become prominent. Episodes of hyperventilation and apnoea may develop [61, 62]. Autosomal recessive forms of PHS (Pitt-Hopkins-like syndrome 1 and 2) are caused by deletions and disease causing variants in the genes *CNTNAP2* and *NRXN1*. Breathing anomalies, epilepsy and autistic features are prominent features in these cases [63, 64]. Another Angelman-like condition is the X-linked Christianson syndrome caused by disease causing variants in the *SLC9A6* gene [65, 66]. Specific characteristics to look for in this condition are eye movement abnormalities, a slim body habitus and an unusually fast EEG rhythm. Furthermore, haploinsufficiency of the *MEF2C* gene has been described due to larger deletions as well as disease causing variants in patients with intellectual disability, absence of speech and seizures. Additionally, stereotypic movements were present [67, 68].

Several chromosome abnormalities have phenotypes that overlap with AS. The most common are the 1p36 subtelomeric deletion, a microdeletion of 17q21, and a terminal deletion of 22q13 [69, 70]. Xq28 duplication including the *MECP2* gene may also present with a phenotype suggestive of AS in males [71]. Profound neonatal hypotonia, the presence of constipation and Rett-like features distinguish Xq28 duplication from AS patients [72]. Several patients with 2q23.1 microdeletions encompassing the methyl binding domain gene *MBD5* and a clinical and behavioural phenotype reminiscent of AS were reported [73]. Seizures, ataxia and sleep disturbance were common findings in this group of patients. Microarray studies are therefore clearly indicated in patients with AS-like features.

Some rare metabolic disorders may present with AS-like symptoms. Methyltetrahydrofolate reductase (*MTHFR*) deficiency and adenylosuccinate lyase deficiency have been reported as presenting with learning disability, ataxia, seizures, autistic features and excessive laughter [74, 75]. With MTHFR deficiency homocystinuria is present and treatment with folic acid and betaine may alleviate, though not completely cure symptoms.

Haploinsufficiency of *HERC2*, *ATRX*, *FOXG1, STBPX1* and *SYNGAP1* show some overlapping phenotypic features with AS [70, 76, 77].

Lastly, AS patients with mosaic ID can lack the typical features like absence of speech, ataxia and happy demeanour but can instead present with features typical for PWS like hypotonia, obesity and developmental delay [78].

### Internal and external quality control

The EMQN recommends that all laboratories offering molecular genetic testing for PWS and AS must follow established good laboratory practice, as documented for example in Guidelines for Quality Assurance in Molecular Genetic Testing, published by the Organisation for Economic Co-operation and Development [79].

In addition to following such guidelines, a laboratory should ideally demonstrate that it complies with internationally recognised standards for laboratory testing (e.g. ISO standards 15189: 2012 Medical laboratories –requirements for quality and competence), by achieving formal accreditation with a member organisation of the International Laboratory Accreditation Cooperation (ILAC) or equivalent national accreditation body.

All tests should be validated/verified in individual laboratories prior to implementation; it is not acceptable to rely on the validation of a test by another laboratory, since that does not guarantee that it will perform accurately and reliably in all labs. A series of control samples representing all disease causing variant types should therefore be collected by each laboratory to facilitate test validation/verification, and exchange of samples between laboratories is encouraged to allow this. External quality assessment (EQA) schemes provide further validation of testing procedures and methods, and laboratories should participate annually in appropriate EQA schemes for PWS and AS testing. If this is not possible, inter-laboratory exchange of samples should be arranged to compare and validate test results.

## Discussion/conclusion

This is an update of the practical set of molecular genetic testing and reporting guidelines that have been developed for PWS and AS by Ramsden and colleagues [1]. The update takes into account new developments in terms of techniques, differential diagnoses and reporting standards.

Feedback has been obtained from participants of the 2018 PWS/AS EMQN external quality assessment scheme (105 laboratories from 29 countries). Nine comments were received; most were minor largely typographic corrections and some points of clarity. There was no disagreement on the recommendations made. All comments have been incorporated into this final document.

## Supplementary information

Supplementary Figure 1

Supplementary Figure 2

Supplementary information
