# Supplementary figures and images for: Update of the EMQN/ACGS best practice guidelines for molecular analysis of Prader-Willi and Angelman syndromes
Source: Eur J Hum Genet. 2019 Jun 24;27(9):1326–40. doi: 10.1038/s41431-019-0435-0 (PMC6777528; doi:10.1038/s41431-019-0435-0)

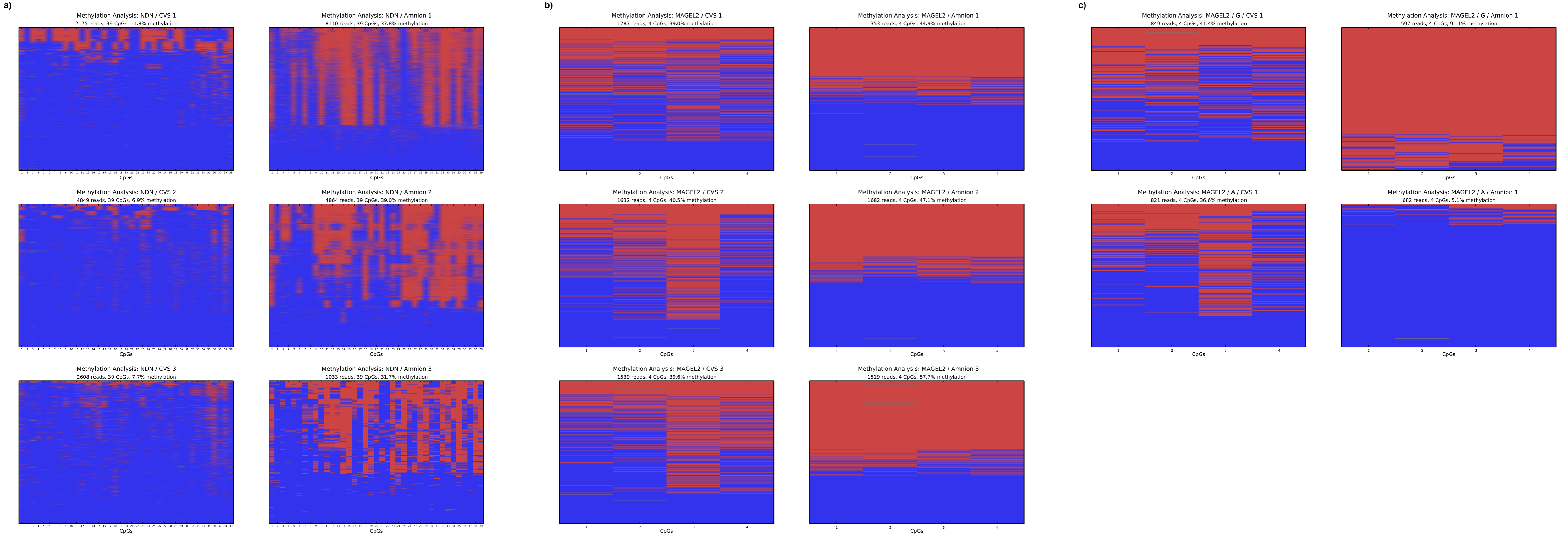

Supplement: Supplementary file 1 — Supplementary Figure 1 [file 41431_2019_435_MOESM1_ESM.pdf]

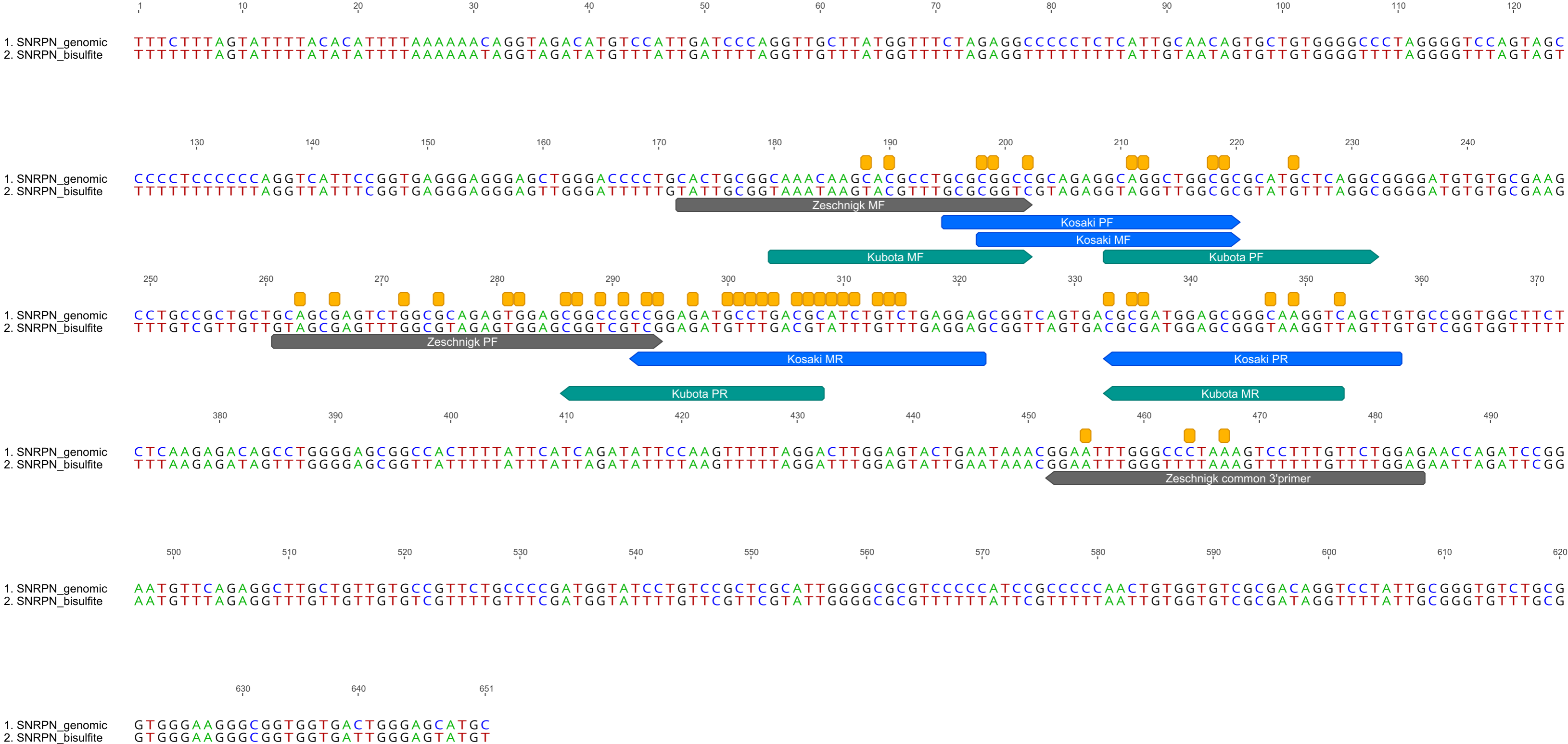

Supplement: Supplementary file 2 — Supplementary Figure 2 [file 41431_2019_435_MOESM2_ESM.pdf]
