# Supplementary material for: Update of the EMQN/ACGS best practice guidelines for molecular analysis of Prader-Willi and Angelman syndromes
Source: Eur J Hum Genet. 2019 Jun 24;27(9):1326–40. doi: 10.1038/s41431-019-0435-0 (PMC6777528; doi:10.1038/s41431-019-0435-0)
Supplement: Supplementary file 3 — Supplementary information [file 41431_2019_435_MOESM3_ESM.docx]

**Supplementary Figure 1: Methylation status of a) *NDN* and b-c) *MAGEL2* in chorionic villi and amniotic fluid samples.**

The methylation status of the *NDN:TSS-DMR* and the *MAGEL2:TSS*-DMR have been investigated by next generation bisulfite sequencing in three chorionic villi (CVS 1-3) and three cultured amniotic fluid samples (Amnion 1-3) (Kanber et al. 2009, Beygo et al. 2013). Lines represent single reads, columns represent investigated CpGs. Blue depicts unmethylated, red methylated CpGs. The number of reads analysed and the overall mean methylation is given above the plots.

a) Plots show the methylation status of the *NDN:TSS*-DMR in three CVS samples (left) and three cultured amniotic fluid samples (right).

b) The methylation status of the *MAGEL2:TSS*-DMR is given for three CVS samples (left) and three cultured amniotic fluid samples (right).

c) Exemplary results of the methylation status of the *MAGEL2:TSS*-DMR after separation of the alleles. By utilising a single nucleotide polymorphism (rs850818 G/A) present in the analysed region reads were assigned to the respective allele. Results for one informative CVS sample (left) and one informative amniotic fluid sample (right) are shown.

**Supplementary Figure 2:** Alignment of the genomic and bisulfite sequence of the *SNRPN* exon 1/promoter region (chr15:24,954,713-24,955,363 (plus strand); UCSC Human Genome Browser; hg38). Single nucleotide polymorphisms (SNPs; based on dbSNP 150) at primer binding sites are indicated as yellow boxes. Primer binding sites for different PCR assays are shown as arrows (Kubota et al. 1997, Kosaki et al. 1997, Zeschnigk et al. 1997).

MF, forward primer for the maternal methylated allele; PF, forward primer for the paternal unmethylated allele; MR, reverse primer for the maternal methylated allele; PR, reverse primer for the unmethylated paternal allele. Note that the bisulfite sequence only shows the methylated allele.

**Supplementary references:**

Beygo J, Ammerpohl O, Gritzan D, Heitmann M, Rademacher K, Richter J, Caliebe A, Siebert R, Horsthemke B, Buiting K. 2013. Deep bisulfite sequencing of aberrantly methylated loci in a patient with multiple methylation defects. PLoS One 8(10):e76953.

Kanber D, Giltay J, Wieczorek D, Zogel C, Hochstenbach R, Caliebe A, Kuechler A, Horsthemke B, Buiting K. 2009. A paternal deletion of MKRN3, MAGEL2 and NDN does not result in Prader-Willi syndrome. Eur J Hum Genet 17(5):582-90.

Kosaki K, McGinniss MJ, Veraksa AN, McGinnis WJ, Jones KL. 1997. Prader-Willi and Angelman syndromes: diagnosis with a bisulfite-treated methylation-specific PCR method. Am J Med Genet 73(3):308-13.

Kubota T, Das S, Christian SL, Baylin SB, Herman JG, Ledbetter DH. 1997. Methylation-specific PCR simplifies imprinting analysis. Nat Genet 16(1):16-7.

Zeschnigk M, Lich C, Buiting K, Doerfler W, Horsthemke B. 1997. A single-tube PCR test for the diagnosis of Angelman and Prader-Willi syndrome based on allelic methylation differences at the SNRPN locus. Eur J Hum Genet 5(2):94-8.
